# Unlocking Creative Movement with Inertial Technology

**DOI:** 10.3390/brainsci15090922

**Published:** 2025-08-26

**Authors:** Eva Sánchez Martz, Alejandro Romero-Hernandez, Beatriz Calvo-Merino, Santiago Fernández González

**Affiliations:** 1 Experimental Psychology Department, Complutense University of Madrid, 28223 Madrid, Spain; sanferna@ucm.es; 2 Software Engineering and Artificial Intelligence Department, Computers Science School, Complutense University of Madrid, 28223 Madrid, Spain; alerom02@ucm.es; 3 Department of Psychology and Neuroscience, Center for Clinical Social and Cognitive Neuroscience, City St. George’s, University of London, London EC1V 0HB, UK

**Keywords:** mental representation, imagery, cognitive performance, inertial sensors, movement analysis, dance creation, cognitive neuroscience

## Abstract

Background: This study examined the influence of creative thinking, shaped by different forms of episodic mental representations, on human movement. The primary objective was to investigate how creativity, elicited through distinct cognitive stimuli, affects movement variability. Methods: Twenty-four professional dancers developed two original dance phrases, each inspired by either a visual or a narrative mental representation. Movement data were collected via inertial sensor technology and subsequently analysed to determine differences in motor expression. Results: The results indicated that movements performed under narrative representation conditions exhibited significantly increased risk-taking behaviour, greater movement amplitude, and a higher overall movement volume compared to those guided by visual stimuli. Conclusions: These findings underscore the role of creativity in modulating both the expressive and physical dimensions of human movement. Moreover, this research demonstrates the potential of inertial sensor technology not only to capture kinematic patterns but also to provide insight into the deeper layers of human artistic and cognitive processes.

## 1. Introduction

This study investigated the potential of inertial sensors to enhance the understanding of the artistic creation of dance movements, with particular emphasis on the relationship between choreographic strategies and the resultant movement patterns.

Inertial sensors have emerged as versatile and reliable tools owing to their capacity to capture precise biomechanical data and their adaptability across diverse environments [1,2]. These sensors have been increasingly utilised in research across a broad spectrum of fields, ranging from clinical studies of movement disorders to the development of novel motor function assessments to sports sciences and human behaviour analysis [3,4,5,6]. More recently, cognitive sciences have also employed inertial sensors to investigate internal action representations and the interactions between cognitive and kinematic mechanisms in human–robot interaction. Recent advances in both cognitive science and embodied technologies have underscored the potential of integrating sensor-based methodologies with artistic practices [7]. Nevertheless, the impact of the nature of mental representations on creative movement processes remains insufficiently explored [8,9]. This study sought to address this gap by examining how cognitive approaches and inertial sensor technologies can be jointly utilised to investigate the role of internal representations in motor creativity. In so doing, we attempted to build upon and extend previous research by combining embodied cognitive theory with sensor-based methodologies within the context of creative movement. This interdisciplinary approach not only advances our understanding of the cognitive mechanisms underpinning motor improvisation but also offers novel empirical insights into how internal representations influence creative motor behaviour.

Creative behaviour is facilitated by the formation of perceptual and associative links between internal representations. This process enables the organisation and integration of information, ultimately resulting in a creative output [10,11]. This broad understanding of creativity encompasses the integration of sensory information with abstract cognition, thereby distinguishing it from neural patterns associated with other advanced cognitive functions [12]. In the context of creativity combined with imagery, it is important to note that cognitive general imagery involves the deliberate generation of mental representations from a first-person perspective [13]. Such representations evoke sensations or ideas related to movement through the use of metaphor. Notably, imagined episodic mental representations preserve the spatiotemporal structure of their referent domains [14,15]. This aspect is of particular importance when considering movement creation initiated through internal representations.

Another pivotal concept within the discipline of movement, creativity, and choreography is that of movement variability [16,17]. This concept is grounded in the principle and capacity to adapt to dynamic contexts. Within this framework, motor creativity entails discovering novel ways to act adaptively or modifying actions to suit new situations, with creative actions being inherently purposeful [18]. Dance provides a distinctive model for investigating the role of mental imagery and internal representations in motor performance and creative cognition. It offers a valuable context for scientific inquiry into the dynamic interplay between cognitive simulation, sensorimotor execution, and the emergence of creative behaviour [19,20,21].

With this in mind, the present research examined how different types of episodic mental representations—induced under two distinct conditions based on the use of visual-episodic imagery versus narrative-episodic imagery—influence creative processes in dance and interact with movement variability. Furthermore, this study aims to elucidate how internal representations contribute to motor creativity by analysing data obtained from inertial sensors affixed to dancers during creative movement tasks. Specifically, it investigates how three variables—movement amplitude, risk-taking behaviour, and overall movement quantity—are modulated throughout the improvisational process, thereby providing insights into the embodied dynamics of creative motor performance. These variables were selected for their potential relevance to the complexity and dynamics of individual–environment interactions, as well as their applicability to everyday movements in non-professional populations [22].

## 2. Method

### 2.1. Participants

Twenty-four professional dancers participated in this study, with a mean age of 30.16 years. All participants had received over 10 years of training and were recruited in collaboration with the Spanish National Dance Company and the Spanish National Ballet. The study sample consisted exclusively of professional dancers currently under active contract with companies affiliated to the National Institute of Performing Arts and Music (INAEM) in Spain, specifically the Ballet Nacional de España and the Compañía Nacional de Danza. Inclusion criteria required formal employment within these institutions and possession of advanced, superior-level dance education. Individuals lacking professional affiliation with the aforementioned companies or without documented higher education in dance were categorically excluded from participation. This stringent selection protocol was implemented to ensure homogeneity and methodological rigour within the study cohort. Ethical approval was granted by the Committee on the Use of Human Subjects at Complutense University of Madrid. (E_20231013_21_EXP, 13 October 2023).

### 2.2. Design and Instruction

A repeated-measures design was employed to investigate the effects of two distinct mental representation tasks—narrative-episodic and visual-episodic—on three motor performance variables: movement amplitude (A), total movement (TM), and risk assumed (R). In the Episodic Visual Representation task, participants were instructed to imagine a place associated with a specific experience and to describe this place through movement, focusing on its structural elements and the functions of objects, with particular emphasis on visual imagery. In the Narrative Representation task, participants imagined the same place as in the Visual Representation condition but were asked to convey it through movement based on the experiences and emotions associated with the space, thereby communicating a narrative. Both conditions were alternated intermittently throughout the experiment.

### 2.3. Sensors and Motion Measurement Measures

The experiment was conducted at the headquarters of the National Dance Company of Spain. Movement data were collected using Perception Neuron’s inertial motion capture technology (see validation at [23]). The 17 inertial measurement unit (IMU) sensors were placed according to the official placement guidelines provided by the Perception Neuron Smartsuit user manual. Specifically, sensors were located on the following anatomical landmarks: head, upper neck, lower neck (T1), upper chest (sternum), lower spine (L5), left and right upper arms, forearms, hands, upper legs (thighs), lower legs (shanks), and feet.

All sensor placements were performed by the same experienced operator, Dr. Alejandro Romero-Hernández, PhD, from the Software Engineering and Artificial Intelligence Department, Computer Science School, Complutense University of Madrid, Spain. His consistent involvement across all testing sessions ensured control over intra-tester variability.

Although the manufacturer does not provide formal intra-tester reliability data, previous studies have reported good reliability of the Perception Neuron system in test–retest settings [24,25], supporting the methodological consistency of the protocol used in this study.

After confirming that all 17 inertial measurement units (IMUs) were powered, correctly assigned, and transmitting stable signals within the Axis Studio software environment, the participant was instrumented with the motion capture suit in a controlled, low-interference setting. Calibration was conducted using the standard multi-pose protocol, comprising three sequential postures: the T-pose (with arms extended horizontally), the A-pose (standing naturally with arms relaxed at the sides), and the S-pose (a semi-squat position with arms fully extended forward). These calibration poses enable the system to align each sensor’s orientation with the participant’s anatomical structure and the global coordinate system. The calibration process was initiated via the software interface and subsequently validated through the 3D avatar preview, where correct sensor alignment and minimal drift were visually confirmed, ensuring an accurate and reliable setup.

**Figure 1:**
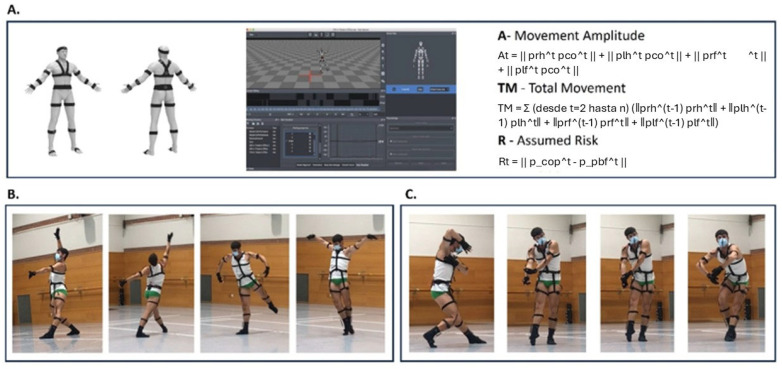
Procedure schema (**A**) Perception Neuron Smart Suit Inertial Sensors. (**B**) Example of dancer creating movement under Narrative Representation Instructions. (**C**) Example of dancer creating movement under Visual Representations Instructions.

These sensors capture full-body movement, transmitting data to the Axis Studio software, which converts it into a skeletal avatar for analysis. For this study, animations were exported in biped (.bvh) format for subsequent data analysis (Figure 1).

The variables, movement amplitude (A), total movement (TM), and assumed risk (R), were measured using inertial sensor data. These metrics were calculated based on the motion of each tracking point and reported in centimetres. This procedure is well established in human movement research using inertial motion capture and has been recognised as a validated tool for this purpose [26,27,28,29].

The Amplitude of Movement (A) is a quantitative measure that represents the cumulative distance travelled by all four limbs—right hand, left hand, right foot, and left foot—relative to a central reference point, known as the centre of gravity. It is calculated as the sum of the norms of the displacement vectors of each limb relative to the centre of gravity at a given time *t*. This formulation quantifies the total displacement of the limbs with respect to the centre of gravity over time, providing a measure of movement amplitude:

At = || prh^t pco^t || + || plh^t pco^t || + || prf^t pco^t || + || plf^t pco^t ||

where

prh^t represents the position of the right hand at time *t*;
plh^t represents the position of the left hand at time *t*;
prf^t represents the position of the right foot at time *t*;
plf^t represents the position of the left foot at time *t*;
pco^t represents the position of the centre of gravity at time t;
‖px^t pco^t‖ denotes the Euclidean distance between limb segment and the centre of gravity at time *t*.

The *Total Movement* (TM) is a metric that quantifies the overall amount of movement performed by an individual by summing the distances travelled by each limb (hands and feet) during a task, calculated as the sum of the norms of the displacement vectors of each limb between two consecutive time steps, *t − 1* and *t*. This formulation quantifies the total movement of all four limbs by summing their respective displacements between consecutive time frames over the entire observation period.

TM = Σ (desde t = 2 hasta n) (‖prh^(t − 1) prh^t‖ + ‖plh^(t − 1) plh^t‖ + ‖prf^(t − 1) prf^t‖ + ‖plf^(t − 1) plf^t‖)

where

prh^t represents the position of the right hand at time *t*;
plh^t represents the position of the left hand at time *t*;
prf^t represents the position of the right foot at time *t*;
plf^t represents the position of the left foot at time *t*;
||px^t − px^(t − 1)|| denotes the Euclidean distance travelled by limb x from time *t* − *1* to *t.*

The Risk Assumed (R) evaluates movement stability by measuring the deviation of the centre of gravity projection (COP) from the midpoint between the feet (PBF), with larger deviations indicating greater instability, and is defined as the norm of the displacement vector between the centre of pressure and the posterior base of support at a given time *t.* This formulation quantifies postural stability by measuring the displacement between the projected centre of gravity and the base of support over time.

Rt = || p_cop^t − p_pbf^t ||

where

p_cop^t represents the position of the centre of gravity projection (COP) at time *t*, which is the point on the ground where the body’s centre of gravity is projected;
p_pbf^t represents the position of the posterior base of support (PBF) at time *t*, which is the midpoint between the left and right feet;
|| p_pbf^t − p_cop^t || denotes the Euclidean distance between the COP and the PBF at time *t*.

In addition, we introduced an “increment” index (inc), representing the difference in performance between Narrative Representation task and Visual Representation task. For example, the mean inc_a of 0.034 indicates an average increase of 34 thousandths of a centimeter in amplitude from Visual Representation task to Narrative Representation task. Negative values for increments suggest that some individuals achieved larger outcomes in Visual Representation, though the overall trend is positive across all variables, indicating greater performance in Narrative Representation task (Table 1).

### 2.4. Statistical Analysis

We used the Shapiro–Wilk test to assess normality for the variables amplitude (A), total movement (TM), and risk assumed (R). Task instruction differences were compared using a paired *t*-test for normally distributed data (amplitude) and the Wilcoxon Signed-Rank test for non-normally distributed data (total movement and risk assumed). The significance level (alpha) was set at 0.05.

## 3. Results

**Figure 2:**
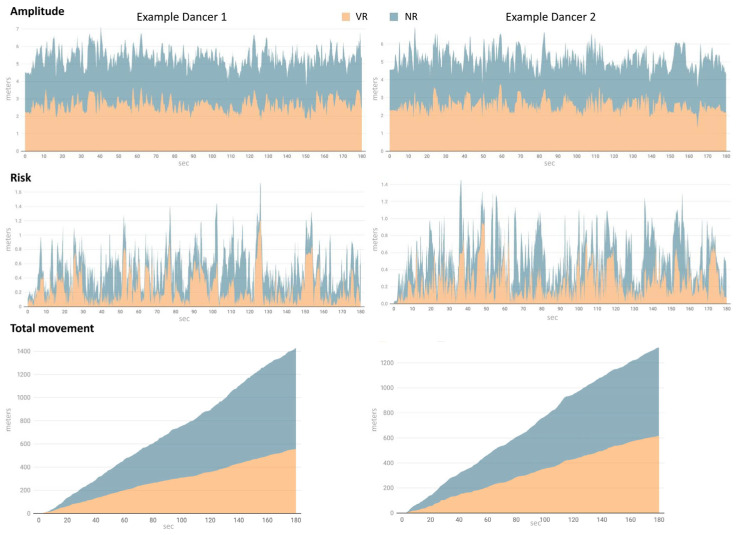
Performance diagrams for two dancers across the Visual Representation (VR) and Narrative Representation (NR) conditions. The *x*-axis represents time (in seconds), and the *y*-axis represents movement amplitude (in meters).

Results revealed significant increases across all three variables when comparing the Visual Representation Condition with the Narrative Representation Condition. Significant differences were found in amplitude (*t*_23_ = 2.73, *p* = 0.012), risk (*Wilcoxon*, *S* = 92, *p* = 0.0057), and total movement (*Wilcoxon*, *S* = 68, *p* = 0.0497), indicating significant measurable effects of the condition on postural parameters (Table 2, Figure 2). Figure 2 illustrates the values obtained for two representative dancers in the Visual Representation (VR) and Narrative Representation (NR) conditions across the three variables: amplitude, risk, and total movement. The diagrams display a clear trend of increased values in the NR condition compared to the VR condition, consistent with the statistical findings reported in Table 2. For both dancers, the amplitude and total movement measures visibly increase in the NR condition, while the risk variable also shows a consistent upward pattern. This graphical evidence reinforces the conclusion that narrative instructions elicit greater postural adjustments, highlighting the significance of the measured differences. The illustrations further emphasise individual variability, with both participants demonstrating consistent directional changes despite differences in magnitude. Importantly, although the mean differences between each condition were small, the consistently positive direction across most participants contributed to the high statistical significance of the findings.

## 4. Discussion

Despite increasing interest in the use of sensor-based technologies in movement research, the role of internal mental representations in shaping creative motor behavior remains insufficiently explored. While inertial sensors have proven effective in capturing biomechanical data across clinical, athletic, and cognitive domains, their integration with cognitive frameworks—particularly in the context of artistic creation—has received limited empirical attention. This study addresses that gap by examining how different types of episodic mental imagery (visual vs. narrative) influence movement variability and creativity during improvisational dance. The rationale for this investigation lies in the need to better understand the embodied mechanisms underlying motor creativity, specifically how internal representations modulate key dimensions of movement. Accordingly, the primary purpose of the study was to assess the effects of these distinct mental representations on amplitude, risk-taking behavior, and overall movement quantity, using inertial sensor data as an objective measure of postural and kinematic change.

We manipulated the nature of the cognitive representations employed by the dancers to inspire and influence the creation of dance movements. Two types of episodic memories were elicited through mental imagery: one emphasising visual content, and the other centred on narrative content. We found significant differences between the two types of imagery in three movement variables measured with inertial sensors during the dance performance: movement amplitude, risk-taking, and overall movement.

Our findings indicate that mental imagery appears to exert a direct influence on the creation of motor outcomes, with distinct effects depending on the type of cognitive representation employed. Specifically, 18 of the 24 participants demonstrated increased movement amplitude during narrative episodic mental imagery, while 19 exhibited a greater propensity for risk-taking under the same condition. Total movement also increased in 14 participants during the narrative condition, although this effect was less pronounced than for the other variables. The consistent increase across all three variables in the narrative condition compared to the visual condition emphasises the significant impact of mental representations on motor execution, particularly within the artistic processes related to creativity.

The integration of inertial technology with dance and creativity raises several important considerations. Firstly, timing flexibility: The methodology employed in this study provided a robust framework for analysing movement amplitude, risk-taking, and total movement over a three-minute dance sequence, with potential for adaptation to longer durations. This temporal dimension is crucial in the performing arts, and dance in particular, which fundamentally unfolds over extended periods [30,31,32]. Secondly, challenges inherent to the use of inertial sensors—such as calibration issues, magnetic disturbances, and sensor biases—must be addressed in study design to mitigate potential artefacts. In our research, professional dancers were given explicit instructions to avoid impact movements against the ground in order to prevent signal loss at any tracking points. Two cases, representing 7.69% of the initial sample, were excluded for this reason, as data cleaning revealed that one or more tracking points ceased registering movement during the sequence. Future research should endeavour to refine sensor technologies to overcome these limitations and extend the application to high-impact movements commonly observed in dance, such as large jump landings, rolling motions, and ground falls.

This study makes a significant contribution to the current body of knowledge in embodied creativity research and human movement by providing a measure of the connection between the creative process and human action. Its impact is twofold. Firstly, it offers a general framework that strengthens the links between technology and creativity [33,34], enabling, for example, the quantification of motor acts within a creative context. Secondly, it contributes specific tool knowledge that facilitates the depiction and understanding of various elements of cognitive representations [35] and how these elements interact with motor output, both generally and in the arts in particular. By demonstrating that representations based on narrative episodic imagery lead to greater movement amplitude, risk-taking, and total movement compared to those based on visual imagery, this research deepens our understanding of how internal representations physically interact with the environment. Such insights hold potential applications beyond cognitive science, including artistic pedagogy and creative practice.

Finally, the analysis of inertial data is advancing rapidly. While the current study focused on movement amplitude, risk, and total movement, further investigation into variables such as movement density or volume could yield additional insights and inspire future research. Moreover, the specific processing and analysis of data driven by artificial intelligence (AI) systems capable of analysing inertial data in real time and making instantaneous decisions are gaining momentum, offering novel opportunities for application in fields where movement analysis and motor action prediction are essential. Exploring these effects in non-artistic contexts and among individuals without dance experience could further broaden their applicability, particularly in clinical settings.

### Possible Limitations

While this study offers valuable insights into the relationship between creative mental representations and human movement using inertial sensor technology, certain limitations should be acknowledged. First, the sample size, although carefully controlled and homogeneous, was limited to 24 professional dancers from specific companies, which may restrict the generalisability of the findings to broader populations or different skill levels. Second, the experimental design focused on only two types of mental representations (visual and narrative), which may not encompass the full spectrum of cognitive processes involved in creative movement. Lastly, the reliance on inertial sensor data, while providing detailed kinematic information, may not capture other dimensions of creativity, such as emotional or motivational factors, that could also influence movement variability. These limitations highlight opportunities for future research to expand the sample diversity, explore additional cognitive constructs, and integrate multimodal assessment methods.

## 5. Conclusions

This study’s results provide novel insights into how distinct episodic mental representations—visual versus narrative—shape creative motor behavior, as objectively measured through inertial sensor technology. The findings demonstrate that narrative mental imagery significantly enhances movement amplitude, risk-taking behavior, and overall movement volume in professional dancers, underscoring the intricate link between cognition and embodied creativity. By integrating cognitive frameworks with biomechanical data, this work contributes to a deeper understanding of the mechanisms underlying creative action, particularly in artistic contexts.

Despite certain limitations, the methodological approach establishes a robust foundation for future research, with promising implications for diverse fields such as artistic pedagogy, cognitive science, and clinical applications. The study highlights the potential of inertial sensor technologies to move beyond purely kinematic analysis toward capturing intrinsic human elements like creativity and decision-making in real-time. Further exploration of additional movement parameters and integration of advanced analytical tools, such as artificial intelligence, will likely expand the applicability and depth of this line of inquiry.

In summary, the present work advances the understanding of the interplay between mental imagery and motor output, opening new avenues for both theoretical development and practical innovation in the study of human creativity and movement.

## Figures and Tables

**Table 1:** Results of the Increment Index. Total Mov = total movement.

Increment	Frequency	Percent
Amplitude < 0	6	25.00%
Amplitude > 0	18	75.00%
Risk < 0	5	20.83%
Risk > 0	19	79.17%
Total Mov < 0	10	45.83%
Total Mov > 0	14	58.33%

**Table 2:** Statistical results for amplitude, risk, and total movement during Visual Representation task and Narrative Representation task.

Variable	N	Mean	Std. Dev.	Min	Median	Max
A_VR	24	2.662	0.134	2.376	2.664	2.892
A2_NR	24	2.696	0.119	2.364	2.708	2.918
R1_VR	24	0.252	0.061	0.138	0.244	0.388
R2_NR	24	0.279	0.058	0.127	0.283	0.433
TM1_VR	24	219.620	79.167	69.080	232.460	357.050
TM2_NR	24	266.150	87.835	65.700	284.790	449.870

Note: A = amplitude, R = risk, and TM = total movement. VR refers to Visual Representation task and NR to Narrative Representations task. N = sample size; Std. Dev. = standard deviation; Min = minimum value; Max = maximum value. All measurements presented are expressed in centimeters (cm).

## Data Availability

Data is available in https://figshare.com/s/abddd08838e1fef5e928 (accessed on 25 July 2025).
